# Exploring the Multi-Target Performance of Mitochondriotropic Antioxidants against the Pivotal Alzheimer’s Disease Pathophysiological Hallmarks

**DOI:** 10.3390/molecules25020276

**Published:** 2020-01-09

**Authors:** Sofia Benfeito, Carlos Fernandes, Santiago Vilar, Fernando Remião, Eugenio Uriarte, Fernanda Borges

**Affiliations:** 1 CIQUP/Department of Chemistry and Biochemistry, Faculty of Sciences, University of Porto, R. Campo Alegre 1021/1055, 4169-007 Porto, Portugal; cevinhas@gmail.com; 2 Departmento Química Orgánica, Facultad de Farmacia, Universidad de Santiago de Compostela, Campus Vida, 15782 Santiago de Compostela, Spain; qosanti@yahoo.es (S.V.); eugenio.uriarte@usc.es (E.U.); 3 UCIBIO-REQUIMTE, Laboratory of Toxicology, Department of Biological Sciences, Faculty of Pharmacy, University of Porto, R. Jorge de Viterbo Ferreira 228, 4050-313 Porto, Portugal; remiao@ff.up.pt; 4 Instituto de Ciencias Químicas Aplicadas, Universidad Autonoma de Chile, Av. Libertador Bernardo O’Higgins, 7500912 Santiago de Chile, Chile

**Keywords:** Alzheimer disease, mitochondriotropic antioxidants, cholinesterase inhibitors, oxidative stress, β-amyloid, iron accumulation, excitotoxicity

## Abstract

Alzheimer disease (AD) is the most common neurodegenerative disease featuring progressive and degenerative neurological impairments resulting in memory loss and cognitive decline. The specific mechanisms underlying AD are still poorly understood, but it is suggested that a deficiency in the brain neurotransmitter acetylcholine, the deposition of insoluble aggregates of fibrillar β-amyloid 1–42 (Aβ_42_), and iron and glutamate accumulation play an important role in the disease progress. Despite the existence of approved cholinergic drugs, none of them demonstrated effectiveness in modifying disease progression. Accordingly, the development of new chemical entities acting on more than one target is attracting progressively more attention as they can tackle intricate network targets and modulate their effects. Within this endeavor, a series of mitochondriotropic antioxidants inspired on hydroxycinnamic (HCA’s) scaffold were synthesized, screened toward cholinesterases and evaluated as neuroprotectors in a differentiated human SH-SY5Y cell line. From the series, compounds **7** and **11** with a 10-carbon chain can be viewed as multi-target leads for the treatment of AD, as they act as dual and bifunctional cholinesterase inhibitors and prevent the neuronal damage caused by diverse aggressors related to protein misfolding and aggregation, iron accumulation and excitotoxicity.

## 1. Introduction

**Figure 1:**
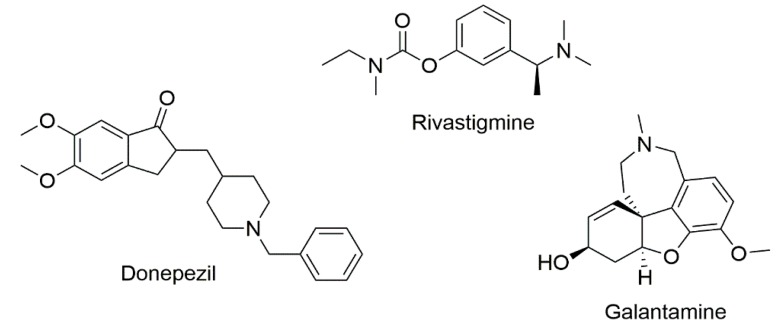
Acetylcholinesterase inhibitors used for the treatment of Alzheimer’s disease.

Alzheimer disease (AD) is a progressive and degenerative neurological disorder resulting in memory loss and cognitive decline. The pathogenesis of AD has been linked to a deficiency in the brain neurotransmitter acetylcholine, a process that is related to the loss of memory and cognitive impairment observed in patients [1,2,3]. Therefore, one of the therapeutic strategies used to slow down the progression of AD symptoms is related to the enhancement of acetylcholine levels, throughthe inhibition of cholinesterases (ChE) located in cholinergic synaptic cleft areas [4,5,6]. In brain synapses, acetylcholine can be hydrolyzed by acetylcholinesterase (AChE) and butyrylcholinesterase (BChE) into choline and acetate [7]. The two major forms of cholinesterases are found in neurons and glial cells, as well as in AD neuritic plaques and tangles [4,8]. Both enzymes have a catalytic anionic subsite (CAS) and a peripheral anionic subsite (PAS) [9,10]. The foremost feature that distinguishes these co-regulators of cholinergic neurotransmission is that BChE can also hydrolyze butyrylcholine, succinylcholine and acetylcholine, although less efficiently than AChE [11,12]. In the healthy brain, AChE is more hydrolytic-specific than BChE [9,13]. However, in AD, the activity of AChE decreases progressively and BChE activity is unchanged or even increased [14,15]. Some studies suggested that BChE plays a key role in maintaining the regulation of cholinergic neurotransmission, compensating for the deficiency in AChE, and thus influence the modulation of motor control, cognition and behavior [16,17,18,19]. Butyrylcholinesterase is considered a promising target for the treatment of later stage cognitive decline in AD. Despite the reduction in AChE activity along the AD process, current therapies are still based on acetylcholinesterase inhibitors (Figure 1). To date, three AChE inhibitors—donepezil (Aricept^®^), rivastigmine (Exelon^®^) and galantamine (Reminyl^®^)—have been approved by the Food and Drug Administration to enhance cholinergic transmission, leading to a mild or moderate improvement in cognitive symptoms [20]. However, none of the current therapies have proven effective to stop the deleterious effects of AD [21,22,23].

The development of selective and potent AChE and BChE inhibitors that can restore acetylcholine normal levels and improve cognitive and memory functions is still an active area in drug discovery. Nevertheless, other causes, like oxidative stress induced by protein misfolded and aggregation, iron accumulation and excitotoxicity, have also been implicated in neuronal death and intrinsically related to this multifactorial neurodegenerative disorder [24,25,26]. The initiation and/or progression of AD pathogenesis has been related with the formation of dense-core plaques, which result from the abnormal extracellular accumulation and deposition of insoluble aggregates of fibrillar β-amyloid 1–42 (Aβ_42_) peptide [27,28], iron accumulation, which can catalyze Fenton-like reactions [29], and the excessive extracellular concentration of glutamate, which can lead to the uncontrolled, continuous depolarization of neurons and trigger a process called excitotoxicity [30,31].

Along our drug discovery program, focused on the development of mitochondriotropic antioxidants, we have already developed cinnamic-based derivatives that act as neuroprotective agents against oxidative stress-induced damage, namely towards 6-hydroxydopamine and hydrogen peroxide [32,33]. Moreover, it was also shown that they have the ability to cross the human cerebral microvascular endothelial (*h*CMEC/D3) cell monolayers (an in vitro BBB model) in a time-dependent manner [32].

**Figure 2:**
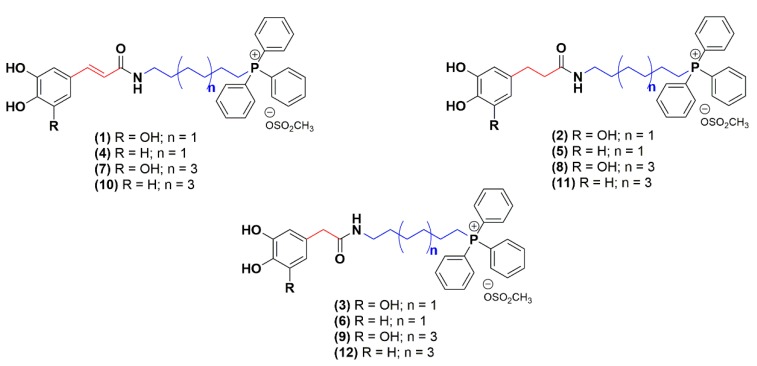
Chemical structures of mitochondriotropic antioxidants used in this work.

The aim of this work was to discover a new multi-target lead compound acting as both antioxidants and ChE inhibitors. Thereby, mitochondriotropic antioxidants (Figure 2) were synthesized de novo and screened toward *ee*AChE (electrophorus electricus) and *eq*BChE (equine serum) enzymes, and their neuroprotective potential toward aggressors related with protein misfolding and aggregation, iron accumulation and excitotoxicity were evaluated in a differentiated human neuroblastoma (SH-SY5Y) cell line. Molecular modelling studies were run using models based on the crystal structure of the target to understand the enzyme–inhibitor interactions. 

## 2. Results and Discussion

### 2.1. Chemistry

In a continuing effort to discovery new multi-target leads for AD, twelve mitochondriotropic antioxidants based on HCA’s scaffold, described in a previous work [32], were synthetized de novo. The structural modifications performed were focused on the aromatic ring substituent pattern (catechol or pyrogallol), on the length of the alkyl linker between carboxamide and triphenylphosphonium moiety (6-(n = 1) or 10-carbon linker (n = 3)) and on the spacer between carboxamide and aromatic ring (A = -CH_2_-; -CH_2_-CH_2_-; -HC = CH-) (Figure 2) [32].

### 2.2. Evaluation of Cholinesterase Inhibitory Activity

The evaluation of the inhibitory efficacy of the mitochondriotropic antioxidants against AChE and BChE was carried out according to the Ellman’s method [34] using donepezil as drug standard. The IC_50_ values for *ee*AChE and *eq*BChE inhibition are shown in Table 1.

Generally, an affinity for *eq*BChE (nanomolar range) over *ee*AChE inhibitory activity was observed for the majority of the mitochondriotropic antioxidants. Compounds **4**, **7** and **10** (Table 1) displayed the highest inhibitory potency towards *eq*BChE when compared to donepezil (IC_50_ = 1.98 µM). The structural modifications performed in the HCA scaffold led to the conclusion that (i) the elongation of the length of the alkyl linker significantly increased the inhibitory activity towards *eq*BChE; (ii) compounds with methylene (*R*’ = -CH_2_-) or ethylene (*R*’ = -H_2_C–CH_2_-) spacer group seems to be less active toward *eq*BChE when compared to the analogues that presented a vinylic spacer and (iii) the number of hydroxyl groups in the phenolic aromatic ring had a slight effect on *eq*BChE inhibitory activity. Generally, compounds bearing a catechol moiety display higher activity toward *eq*BChE.

Regarding *ee*AChE activity, the compounds under study displayed a lower inhibition potency than the standard donepezil, being IC_50_ values higher than 1 µM. On the whole, compounds **5** and **11** with an ethylene spacer exhibited the highest *ee*AChE inhibitory activity, with IC_50_ values of 1.08 and 1.59 µM, respectively (Table 1). Overall, the same tendency found for *eq*BChE inhibitory activity was observed for derivatives with a 10 carbon linker-compounds **9** and **10** that showed a 3 and 2-fold enhancement of *ee*AChE inhibitory activity-when compared to 6 carbon linker analogues (compounds **3** and **4**). Moreover, catechol derivatives **5** and **11** (IC_50_ = 1.08–1.59 µM) were more active than pyrogallol derivatives **2** and **8** (IC_50_ = 5.75–6.76 µM). In contrast, a correlation between the *ee*AChE inhibitory activity and the type of spacer linking the aromatic ring and the carboxamide group was not observed.

### 2.3. Molecular Docking Studies

**Figure 3:**
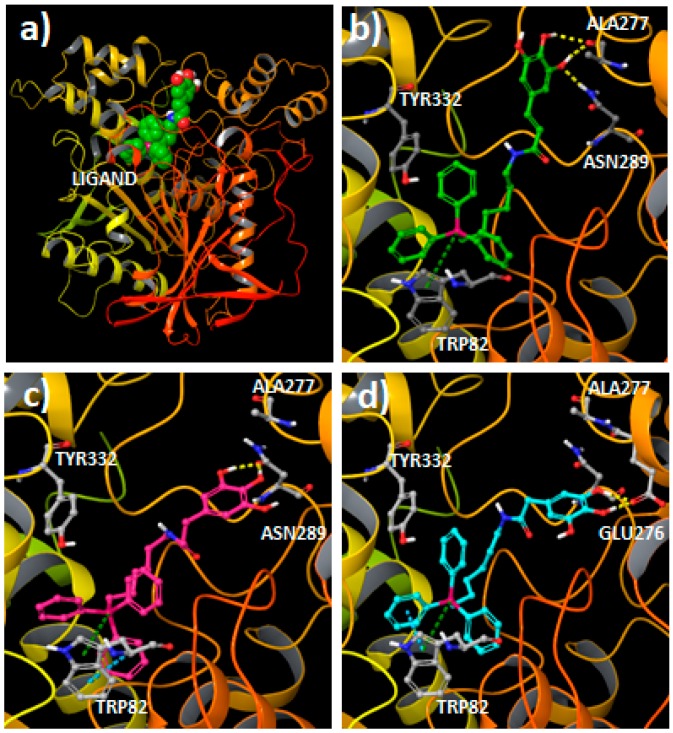
(**a**) General view of compound **1** (CPK representation) inside the BChE (ribbons) extracted from docking simulations; (**b**) Pose generated by docking for compound **1** (green carbons) in the BChE; (**c**) Binding mode extracted for compound **2** (pink carbons) in the BChE; (**d**) Binding mode calculated by docking for compound **3** (color code: hydrogen bonds represented in yellow dashes, green dashes for π–cation interactions, blue dashes for π–π stacking interactions).

As the studied compounds showed greater affinity (lower IC_50_ values) toward *eq*BChE, molecular docking studies were conducted to have a deep insight into the ligand–enzyme interactions, using the crystallized *h*BChE protein structure from the PDB (code 4B0O) [35]. First, the compounds **1**, **2** and **3**, that have the same aromatic substitution pattern and alkyl linker but a different type of spacers, were docked to the protein using Glide SP [36]. Geometrical assessment of the docking protocol was carried out with different co-crystallized ligand structures. Although docking simulations showed some differences in the binding of the derivatives, the results also yielded common interaction features for the different ligands. All the compounds placed the triphenylphosphonium cation in the bottom of the cavity and near the catalytic triad integrated by residues Ser198, Glu325 and His438, whereas the hydroxyphenyl group was directed toward the surface of the pocket (Figure 3).

From the docking simulations, it was observed that the presence of the vinylic spacer in Compound **1** produce some differences in the position of the aromatic moiety when compared to Compounds **2** (ethylene spacer) and **3** (methylene spacer). In fact, Compound **1** established hydrogen bonds with residue Ala277 and Asn289 using the hydroxyl groups of the aromatic ring (Figure 3a,b), and productive π–cation interactions between the triphenylphosphonium group and the residue Trp82 were observed. This type of interaction was described to be relevant to the activity [37,38]. Besides, Compound **1** showed strong Coulomb interactions with residues SBG198 (conjugated Ser in the aged enzyme), Asp70 and Glu197. For Compounds **2** and **3**, a different binding area for the hydroxyphenyl moiety (Figure 3c,d), closer to residues Asn68 and Gln119 was detected. The binding mode for Compound **2** brings forth hydrogen bonds between the phenolic groups and the amide in residue Asn289. Other interactions detected for Compound **2** included π–π stacking and π–cation interactions between the triphenylphosphonium group and the residue Trp82. Compound **3**, with a shorter spacer between the aromatic ring and the amide group, placed the phenolic groups close to the residue Glu276. Hydrogen bonds between 3,4-hydroxyl groups and the residue were detected. Moreover, the compound established π–π stacking and π–cation interactions with the residue Trp82. According to the docking results, the spacer between the carboxamide group and the aromatic ring can determine the position of the hydroxyphenyl framework, with a consequent impact on the interaction of the hydroxyl groups with the protein. Besides, Compound **1** showed an extended binding pose, while Compounds **2** and **3** presented a more compacted aliphatic chain conformation to fit the hydroxyphenyl moiety in the described protein region. Overall the data are in accordance with the experimental data, as it was observed that the introduction of a vinyl spacer between the carboxamide and the aromatic ring increased the BChE activity.

**Figure 4:**
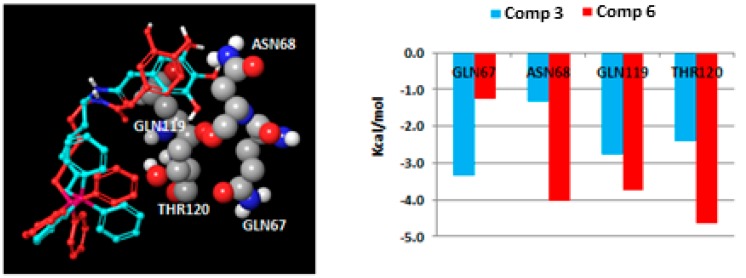
Coulomb and van der Waals interactions between the hydroxyphenyl framework of Compounds **3** and **6** and the BChE residues.

Another significant influence on the protein–ligand interactions resides in the number of hydroxyl groups in the aromatic ring. Compounds bearing a catechol moiety showed an amplified binding when compared to their pyrogallol counterparts. The main difference was found between Compounds **3** and **6** (IC_50_ = 4.52 and 0.93 µM, respectively). As the pose determined by docking is quite similar for both compounds (Figure 4), it was important to calculate the residue contributions to the ligand–protein interaction in a distance of 4 Å from the hydroxyphenyl moiety (sum of Coulomb and van der Waals energies). The main differences in the residue contributions have been found in residues Gln67, Asn68, Gln119 and Thr120. While catecholic Compound **6** interacts strongly with the residues Asn68, Gln119 and Thr120, the incorporation of a third hydroxyl group (Compound **3**) in the aromatic ring moiety causes a loss of interactions with these residues, but enhances the interaction with residue Gln67. 

**Figure 5:**
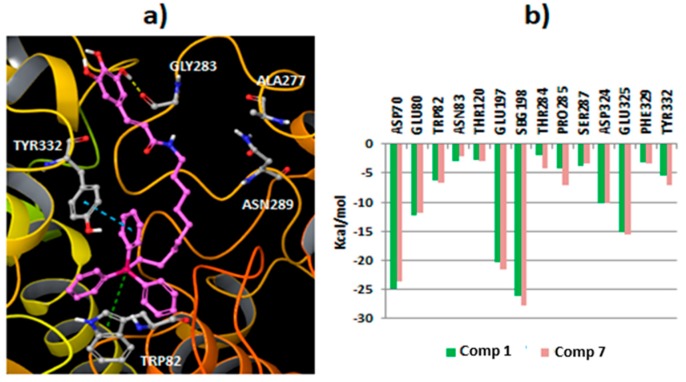
(**a**) Binding mode yielded by molecular docking for Compound **7** inside the BChE (hydrogen bonds: yellow dashes, π–cation interactions: green dashes, π–π stacking interactions: blue dashes); (**b**) Residue contributions (sum of Coulomb and van der Waals energies) to the binding between the BChE and Compounds 7 (magenta color) and 1 (green color).

As the extension of the aliphatic chain between the triphenylphosphonium moiety and the carboxamide is also related to an increase in the BChE activity, additional studies were performed with Compound **7**, the most active compound in the series. This compound positioned the triphenylphosphonium moiety in a similar region to Compound 1 and established π–π stacking interactions with residue Tyr332 and π–cation interactions with residue Trp82. However, as Compound **7** is larger, the hydroxyphenyl group points toward a shallower region in the protein pocket (Figure 5a) and anchored the 3-hydroxyl group with residue Gly283. A comparison of the profiled energy contribution of different residues to the binding of Compounds **1** and **7** is shown in Figure 5b. The main contributions (Coulomb and van der Waals energies) were located in residues SBG198, Asp70, Glu197 and Glu325.

Remarkably, the molecular docking data, which is in accordance with the experimental results, showed that this type of compound is able to interact with both the catalytic active site (CAS) and the PAS of BChE, acting as a bifunctional inhibitor.

### 2.4. Evaluation of Neuroprotective Outline 

The total antioxidant capacity of the mitochondriotropic compounds under study was previously evaluated by measuring their ability to scavenge DPPH and ABTS^+^ radicals [32]. In general, all the compounds presented noteworthy antioxidant activity. Although the variation in the length of the alkyl linker and the type of spacer led to slight changes in antioxidant activity, it was found that the main driving force is related to the aromatic substitution pattern (catechol or pyrogallol). In fact, pyrogallol compounds displayed a higher antioxidant activity than their catechol counterparts, a fact that may be explained by the presence of an extra hydroxyl group and its capacity to stabilize the formed radical.

Following the preliminary screening of antioxidant activity in cell-free systems, the cytoprotective effects of the compounds were evaluated in a human neuroblastoma SH-SY5Y cell line. The differentiated human SH-SY5Y cell line has been widely used as an in vitro model for the evaluation of the neuroprotective capacity of new ligands against a range of oxidative stress inducers [39].

In this work, the neuroprotective potential of the mitochondriotropic antioxidants under study was evaluated toward aggressors (glutamate, iron(III) and Aβ_42_) related with excitotoxicity, iron accumulation and protein misfolding and aggregation, which are deeply related to AD progression and neuronal death [25,40,41]. The cytotoxic profile at the same concentrations has been reported [32].

Experimental evidence suggests that the selected aggressors possess mechanisms of neuronal cell death related to oxidative stress, a relevant feature in the neuropathology of neurodegenerative disorders [42,43]. Glutamate can induce intracellular reactive oxygen species (ROS) accumulation though blockage of the cystine/glutamate antiporter, leading to glutathione depletion and down-regulation of superoxide dismutase activity, and trigger neuronal damage and death [44]. Iron (III) is a pro-oxidant involved in the oxidative damage in neuroblastoma cells, due to its catalytic activity in Fenton reaction [41]. Several lines of evidence indicate that amyloid beta-peptide (Aβ), one of the most important hallmarks in AD, induces oxidative stress. Aβ_40_ and Aβ_42_ represent the most abundant forms in the brain, and their toxicity was associated with the generation of free radicals, that, in turn, promote lipid peroxidation and protein oxidation [40]. 

From results obtained from the ChE inhibition assay, a noteworthy inhibitory effect on *ee*AChE and *eq*BChE was found for Compounds **5** (IC_50_ = 1.08 µM for AChE) and **7** (IC_50_ = 0.11 µM for BChE), respectively. Accordingly, these two compounds were selected for the study as protective agents against glutamate, iron(III) and Aβ_42_. Surprisingly, Compound **5** showed chemical instability, which compromised the study’s feasibility. Alternatively, Compound **11** (IC_50_ = 1.59 µM for AChE) was tested for its protective outline toward the aforementioned insults. From a previous cytotoxicity study, we found that Compound **11** presented cytotoxicity at 50 µM [32], so only non-cytotoxic concentrations were used.

**Figure 6:**
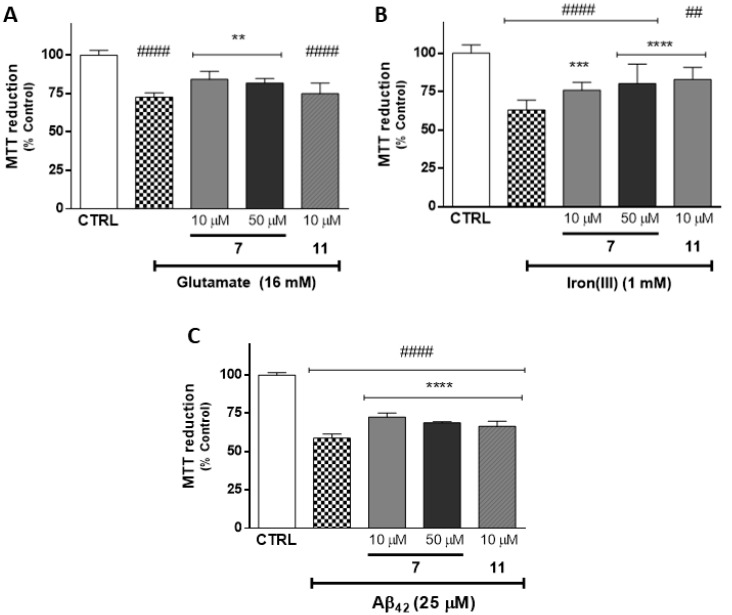
Evaluation of the protective effects of Compounds 7 (10 and 50 µM) and 11 (10 µM) in SH-SY5Y cells against glutamate (16 mM, **A**), iron(III) (1 mM, **B**) and Aβ_42_ (25 µM, **C**) as oxidative stress inducers. The cells were pre-treated with antioxidants for 30 min and exposed to the aggressors for 24 h. The data are expressed as the means of three independent experiments together with the standard deviation (Mean ± SD). Statistical comparisons were estimated using the nonparametric method of Kruskal–Wallis [one-way ANOVA on ranks] followed by Dunn’s post hoc test (**A**,**B**) and using the parametric method of one-way analysis of variance (ANOVA) followed by Dunnett’s multiple comparison test (**C**, F = 364.2) (Supplementary Table S2). In all cases, *p* values lower than 0.05 were considered significant (^##^ *p* < 0.05, ^####^ *p* < 0.0001 vs. control data; ** *p* < 0.01, *** *p* < 0.001, **** *p* < 0.0001 vs. aggressor values).

After 30 min of pre-treatment with Compounds **7** (10 and 50 µM) and **11** (10 µM), the cells were exposed to different aggressors, described above, for 24 h. The results are presented in Figure 6 and in Supplementary Table S1.

Cells treated with glutamate (16 mM, Figure 6A), iron(III) (1 mM, Figure 6B) and Aβ_42_ (25 µM, Figure 6C) caused a significant decrease in cell metabolic activity (*p* < 0.0001) of about 72.7% ± 2.8%, 63.1% ± 6.2% and 58.7% ± 2.8%, respectively, when compared with control cells. As shown in Figure 6A,B, Compound **11** (10 µM) was able to significantly revert the neuronal damage caused by iron(III) and Aβ_42_ (82.9% ± 7.9% and 66.4% ± 3.4%, respectively). For the same concentrations tested, Compound **7** was able to significantly protect neuronal cells after treatment with all stressors. This data suggested that Compound **7** presented greater neuroprotective effects than Compound **11**. The same trend was observed when Compound **7** was tested at 50 µM, which led a significant increase in metabolic activity when compared with cells treated only with glutamate (81.9% ± 2.8%, *p* < 0.0001), iron(III) (80.2% ± 12.8%, *p* < 0.0001) and Aβ_42_ (68.1% ± 0.7%, *p* < 0.0001).

Taking into account the structure of the Compounds **7** and **11**, one can conclude that the enhanced capacity of Compound **7** to prevent neuronal damage is related to the presence of pyrogallol and vinylic moieties.

## 3. Materials and Methods 

### 3.1. Reagents and Apparatus

All reagents used were of analytical grade acquired from Sigma-Aldrich (St. Louis, MO, USA) and TCI Chemicals (Lisboa, Portugal) and used without additional purification. The solvents were pro analysis grade and were acquired from Panreac Química (Barcelona, Spain), Merck (Lisboa, Portugal), Carlo Erba Reagents (Val de Reuil, France) and Sigma Aldrich (St. Louis, MO, USA). Acetylcholinesterase (*ee*AChE), butyrylcholinesterase (*eq*BChE), acetylthiocholine iodide (ATCI), butyrylthiocholine iodide (BTCI) and 5,5′-dithiobis-(2-nitrobenzoate) (DTNB) used in enzymatic assay were from Sigma-Aldrich (St. Louis, MO, USA). Spectrophotometric studies were performed on a BioTek ^®^Synergy HT plate reader.

Concerning the cell-based studies, all reagents used were of analytical grade or of the highest grade available. Differentiated human neuroblastoma (SH-SY5Y) cells were obtained from the American Type Culture Collection (ATCC, Manassas, VA, USA) and their cultures and cell differentiation was performed as previously described by Benfeito et al. [32]. Dulbecco’s Modified Eagle’s Medium (DMEM) with 4.5 g/L glucose, 3-(4,5-dimethylthiazol-2-yl)-2,5-diphenyltetrazolium bromide (MTT), retinoic acid, 12-*O*-tetradecanoylphorbol-13-acetate (TPA) and L-glutamic acid were obtained from Sigma Aldrich (St. Louis, MO, USA). Reagents used in cell culture, such as heat-inactivated fetal bovine serum (FBS), antibiotic (10000 U/mL penicillin, 10000 μg/mL streptomycin), Non-essential amino acids solution (100×, NEAA) and Hanks’ balanced salt solution (HBSS), were purchased from Gibco Laboratories (Lenexa, KS, USA). Aβ-amyloid (1–42) was obtained from GenScript (New Jersey, USA) and dimethylsulfoxide (DMSO) was obtained from Merck (Darmstadt, Germany). 

### 3.2. Chemistry

The synthetic methodologies and spectroscopic characterization data (nuclear magnetic resonance spectroscopy and mass spectrometry) of the compounds have been previously described [32].

### 3.3. Enzymatic Assays

Acetylcholinesterase and butyrylcholinesterase inhibitory activities of the compounds under study were evaluated spectrophotometrically following Ellman’s method [34] using AChE from electrophorus electricus (*ee*AChE) and BChE from equine serum (*eq*BChE). The lyophilized enzymes were dissolved in sodium phosphate buffer (0.25 M, pH 7.4) to make a stock solution of *ee*AChE (1000 U/mL) or *eq*BChE (25 U/mL). The stock solutions of DTNB (2.14 mM) were also performed in a phosphate buffer, while ATCI (1.5 mM in *ee*AChE assay) or BTCI (4.0 mM in *eq*BChE assay) solutions were prepared in deionized water. Briefly, in each well, 100 μL of phosphate buffer, 40 μL of DTNB, 20 μL of the tested compounds (100 *µ*M solution dissolved in 0.1% DMSO and phosphate buffer) and 20 μL of cholinesterase solution were pre-incubated in a 96-well microplate for 5 min at 30 °C. Then, a 20 μL solution containing the enzyme subtract was added and the final absorbance measured at 412 nm for 5 min. The rate of the reaction before adding the enzyme was subtracted from that obtained after enzyme addition in order to correct for spontaneous hydrolysis of substrate. Donepezil, an inhibitor of AChE, and a sodium phosphate buffer were used as the reference compound and control, respectively. The IC_50_ values were calculated by interpolation of dose–response curves using GraphPad PRISM version 6 and referred to as mean with 95% confidence interval (CI 95%).

### 3.4. Molecular Docking Simulations

Molecular docking was carried out using the Schrödinger 2017 package [36]. The BChE protein structure was downloaded from the Protein Data Bank (PDB code: 4B0O) [35]. The module Protein Preparation Workflow [36] was used in the pre-processing of the structure before docking, including different steps, such as the addition of cap termini, hydrogen bond network optimization and generation of suitable protonation states for some residues, among other procedures. Water molecules in the protein pocket were deleted, with the exception of a water molecule that established hydrogen bond interactions with the residues Asp70 and Ser79. A protein grid with a length of 20 Å was calculated using the co-crystallized ligand as a center. Glide SP (Standard Precision) was used to dock the ligands in the pocket [36]. The best ligand pose according to the parameter “Emodel energy” was retained as representative of the simulations and shown for graphical purposes. Validation of the docking protocol was carried out through the calculation of root mean square deviation (RMSD) values between theoretical poses obtained from the docking and co-crystallized ligand structures extracted from the PDB (RMSD values: 4B0O = 1.65, 4AXB = 4.26, 4BDS = 0.37, 1P0M = 3.37, 1P0P = 2.25).

### 3.5. Cellular Culture Conditions

Differentiated human neuroblastoma cells were routinely cultured into 75 cm^3^ flasks and maintained in DMEM high glucose (4.5 g/L glucose), supplemented with 10% heat-inactivated FBS (*v*/*v*), 1% NEAA (*v*/*v*) and 1% penicillin/streptomycin (*v*/*v*). The cell cultures were maintained at 37 °C in a humidified 5% CO_2_–95% air atmosphere and passaged once a week by trypsinization (0.25% trypsin). Neuroblastoma SH-SY5Y cells were seeded onto 96 well plates at a density of 25,000 cells/cm^2^ in cell culture medium with *trans*-retinoic acid (RA, final concentration of 10 µM) and incubated for three days at 37 °C in a humidified, 5% CO_2_–95% air atmosphere. Then, the medium was supplemented with 12-*O*-tetradecanoylphorbol-13-acetate (TPA, final concentration of 80 nM), and cells were incubated for three days. Stock solutions of RA (10 mM) and TPA (160 µM) were prepared in DMSO. To avoid phenotypic changes, the cells used for all experiments were taken between the 19th and 28th passages.

### 3.6. Evaluation of Neuroprotective Outline in SH-SY5Y Cells

The neuroprotective efficacy of mitochondriotropic antioxidants **7** and **11** was evaluated in a cell-based assay against diverse oxidative stress inducers. Briefly, SH-SY5Y cells were pre-treated with Compounds **7** and **11** followed by incubation with the well-described aggressors that play a role in AD progression, namely Aβ-amyloid 1-42 (Aβ_42_) [45], ferric nitrilotriacetate (FeNTA) [41] and glutamic acid [25]. Differentiated SH-SY5Y cells were pre-treated with the test compounds **7** (10 and 50 µM) and **11** (10 µM) for 30 min. Then, a culture medium containing Aβ_42_ (25 µM), iron(III) (1 mM) or glutamic acid (16 mM) was added, and cells were incubated for additional 24 h. A negative control (cells treated with culture medium containing DMSO 0.1%) and a positive control (cells treated with culture medium containing DMSO 0.1%, followed by treatment with aggressors) were also included. Cell viability was estimated using the MTT reduction assay, as described by Fernandes et al. [46].

### 3.7. Statistical Analysis in Cellular Model

The data obtained are expressed as mean ± standard deviation (SD) from three independent experiments performed in triplicate. All statistical analyses were performed using GraphPad PRISM version 6 for Windows. The normality of data distribution was evaluated using three normality tests: KS normality test, D’Agostino and Pearson omnibus normality test and Shapiro–Wilk normality test. For data with a a parametric distribution, statistical comparisons between groups were estimated using the parametric method one-way analysis of variance (ANOVA) followed by Dunnett’s multiple comparison test. For data with a non-parametric distribution, statistical comparisons were estimated using the nonparametric method of Kruskal-Wallis [one-way ANOVA on ranks] followed by Dunn’s post hoc test. In all cases, *p* values lower than 0.05 were considered significant.

The experimenters were not blinded to the treatment groups during data analysis. The researcher that performed the cellular in vitro assays knew the content of each sample and was responsible for the statistical data analysis. 

## 4. Conclusions

Mitochondriotropic antioxidants based on hydroxycinnamic acid have been screened toward ChEs. Generally, the compounds under study displayed affinity for *eq*BChE inhibition in nanomolar range. Structure–activity relationships showed that the elongation of the length of alkyl linker significantly increased the inhibitory activity toward *ee*AChE and *eq*BChE. Additionally, molecular docking simulations toward crystalized human BChE showed that the triphenylphosphonium cation was placed in the bottom of the cavity and near the catalytic triad integrated by residues Ser198, Glu325 and His438, whereas the hydroxyphenyl moiety was directed toward the surface of the pocket. The data pointed out that this type of mitochondriotropic antioxidant behaves as a bifunctional inhibitor. Given the promising results obtained in the ChE inhibitory assay, the neuroprotective outline of Compounds **7** and **11** were further evaluated in differentiated SH-SY5Y cells against glutamate, iron(III) and Aβ_42_ damage inducers. 

Notably, due to its inhibitory activity toward ChE and remarkable neuroprotective properties, the mitochondriotropic antioxidants **7** and **11** can be viewed as multi-target leads that, after optimization, can afford new, disease-modifying AD drug candidates. Due to the multifactorial nature of AD, other targets must be taken into consideration for future work on this class of compounds.

## 5. Patents

Mitochondriotropic antioxidants, processes and applications are under patent (PCT/IB2017/056412; US 2019/0248816 A1). FB is a co-founder of University of Porto spin-off company MitoTAG, but no competing interests exist. 

## Figures and Tables

**Table 1:** Cholinesterases inhibitory activity (IC_50_) data of the mitochondriotropic antioxidants and donepezil.

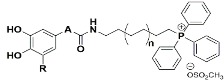				IC_50_ (µM)—Mean (CI 95%) ^1^		
Compound	R	A	n	*ee*AChE ^2^	*eq*BChE ^3^	SI ^4^
**1**	OH	-HC = CH-	1	2.94 (2.58−3.35)	0.41 (0.36−0.47)	7.2
**2**		-H_2_C–CH_2_-		6.76 (6.02−7.59)	3.16 (2.80−3.56)	2.1
**3**		-CH_2_-		8.98 (7.98−10.01)	4.52 (3.52−5.82)	2.0
**4**	H	-HC = CH-	1	6.32 (6.02−6.63)	0.12 (0.11−0.14)	52.7
**5**		-H_2_C–CH_2_-		1.08 (1.00−1.18)	2.52 (1.79−3.53)	0.4
**6**		-CH_2_-		3.88 (3.55−4.23)	0.93 (0.76−1.14)	4.2
**7**	OH	-HC = CH-	3	3.39 (3.03−3.79)	0.11 (0.09−0.14)	30.8
**8**		-H_2_C–CH_2_-		5.75 (5.47−6.04)	0.80 (0.72−0.90)	7.2
**9**		-CH_2_-		3.35 (2.78−4.03)	0.53 (0.43−0.64)	5.7
**10**	H	-HC = CH-	3	3.59 (3.32−3.89)	0.15 (0.12−0.18)	23.9
**11**		-H_2_C–CH_2_-		1.59 (1.47−1.73)	0.32 (0.28−0.40)	4.9
**12**		-CH_2_-		2.44 (2.13−2.79)	0.24 (0.22−0.27)	10.2
**Donepezil**				0.045 (0.039−0.052)	1.98 (1.78−2.19)	---

^1^ CI: confidence interval at 95 %; ^2^ *ee*: electrophorus electricus; ^3^ *eq*: equine serum; ^4^ SI: selectivity index = IC_50_ (*ee*AChE)/IC_50_ (*eq*BChE).

## Supplementary Materials

The following are available online, Table S1: Data are means ± standard deviation (SD) and the results are expressed as % of control (CTRL = 100%) from three independent experiments (n = 3), Table S2: Values obtained from statistical data.
