# Supplementary material for: Exploring the Multi-Target Performance of Mitochondriotropic Antioxidants against the Pivotal Alzheimer’s Disease Pathophysiological Hallmarks
Source: Molecules. 2020 Jan 9;25(2):276. doi: 10.3390/molecules25020276 (PMC7024345; doi:10.3390/molecules25020276)
Supplement: Supplementary file 1 [file molecules-25-00276-s001.pdf]

molecules

Table 1. Data are means ± standard deviation (SD) and the results are expressed as % of control (CTRL = 100%) from three independent experiments (n = 3).

	**Control**	**Glutamate (16 mM)**	**Compound 7**		**Compound 11**
			10 μM	50 μM	10 μM
**MTT reduction (% ctrl)**	100.00 ± 4.83	72.68 ± 2.81	84.24 ± 5.32	81.90 ± 2.81	74.97 ± 6.87
	**Control**	**Iron(III) (1 mM)**	**Compound 7**		**Compound 11**
			10 μM	50 μM	10 μM
MTT reduction (% ctrl)	100.00 ± 5.31	63.06 ± 6.25	75.90 ± 5.25	80.22 ± 12.76	82.87 ± 7.87
	**Control**	**Aβ (25 μM)**	**Compound 7**		**Compound 11**
			10 μM	50 μM	10 μM
MTT reduction (% ctrl)	100.00 ± 1.67	58.73 ± 2.76	72.55 ± 2.67	68.81 ± 0.70	66.43 ± 3.45

Table 2. Values obtained from statistical data.

	**Control**	**Glutamate (16 mM)**	**Compound 7**		**Compound 11**
			10 μM	50 μM	10 μM
	D'Agostino & Pearson omnibus normality test				
K2	1.348	0.7907	6.693	1.545	16.37
P value	0.5096	0.6735	0.0352	0.4618	0.0003
	Shapiro-Wilk normality test				
W	0.9297	0.9052	0.8338	0.9328	0.7429
P value	0.5135	0.2839	0.0492	0.5084	0.0045
	KS normality test				
KS distance	0.9297	0.9052	0.8338	0.9328	0.7429
P value	0.5135	0.2839	0.0492	0.5084	0.0045
	**Control**	**Iron(III) (1 mM)**	**Compound 7**		**Compound 11**
			10 μM	50 μM	10 μM
	D'Agostino & Pearson omnibus normality test				
K2	2.705	5.496	6.409	4.708	0.4683
P value	0.2586	0.0641	0.0406	0.0950	0.7912
	Shapiro-Wilk normality test				
W	0.9199	0.9202	0.8723	0.9112	0.9761
P value	0.0582	0.0034	0.0194	0.0375	0.8153
	KS normality test				

KS distance	0.1943	0.1654	0.2120	0.1491	0.1087
P value	0.0197	0.0025	0.0317	0.1795	0.2000

	**Control**	**Aβ** (25 μM)	**Compound 7**		**Compound 11**
			10 μM	50 μM	10 μM
	D'Agostino & Pearson omnibus normality test				
K2	0.3732	2.045	3.580	0.008512	1.127
P value	0.8298	0.3596	0.1670	0.9958	0.5691
	Shapiro-Wilk normality test				
W	0.9466	0.8570	0.9313	0.9926	0.9465
P value	0.6529	0.0888	0.4935	0.9981	0.6517
	KS normality test				
KS distance	0.1566	0.2676	0.2111	0.1321	0.2078
P value	0.2000	0.0625	0.2000	0.2000	0.2000
